# Reduced mother-to-child transmission rates of HIV between 2017 and 2020 in Kenya. What changed?

**DOI:** 10.4102/jphia.v15i1.626

**Published:** 2024-10-10

**Authors:** Justin Mandala, Linda Muyumbu, Gwyneth Austin, Everline Ashiono, Kayla Stankevitz, Moses Bateganya, Otto Chabikuli

**Affiliations:** 1 HIV Department, FHI 360, Washington DC, United States of America; 2 Department of Strategic Information, FHI 360, Nairobi, Kenya; 3 Research Department, FHI 360, Raleigh, United States of America; 4 Ampath, Nairobi, Kenya; 5 HIV Department, FHI 360, Raleigh, United States of America; 6 Department of TB, United States Agency for International Development (USAID), Dar-es-Salam, Republic of United Tanzania; 7 East and Southern Africa Regional Office, FHI 360, Raleigh, United States of America

**Keywords:** HIV, PMTCT, ART, Kenya, mother, child

## Abstract

**Background:**

In 205 health facilities, mother-to-child transmission (MTCT) of human immunodeficiency virus (HIV) rates were reduced from 7.4% to 2.1% between 2017 and 2020, respectively.

**Aim:**

To determine characteristics that potentially correlate to the change in MTCT rates between two time points.

**Setting:**

Study was conducted in Kenya, semi-urban and rural areas.

**Methods:**

A retrospective, cross-sectional, exploratory analysis of programme implementation at two points in time (2017 and 2020). Between 2017 and 2020, we compared over 170 mother–infant pairs where MTCT occurred to over 6000 mother–infant pairs where MTCT did not occur through the following factors: (1) location of health facilities, (2) mother and infant characteristics, (3) access to antiretroviral therapy (ART), and (4) viral load suppression. Bivariate and multivariable logistic regression models were used to identify factors associated with MTCT.

**Results:**

Factors significantly associated with reduced MTCT rates were time points, mother’s age, infant age at first test, proportions of mothers receiving ART, and maternal viral load. When restricting the analysis to the sub-counties contributing data at both time points, the results were similar; however, counties’ location became significant in the updated model, as did the interaction term for mother and infant receipt of antiretrovirals (odds ratio [OR]: 0.228; *p* = 0.04).

**Conclusion:**

What changed between 2017 and 2020 is a higher proportion of pregnant women living with HIV received ART. Also, unlike in 2017, in 2020, tenofovir disoproxil fumarate was the backbone of the ART regimen for the prevention of MTCT.

**Contribution:**

The findings can potentially inform efforts on elimination of mother-to-child transmission of HIV.

## Introduction

In 2020, an estimated 1.7 million children (0–14 years old) were living with human immunodeficiency virus (HIV) worldwide, and 150 000 were newly infected. The majority of new infections in children (87%) occur in sub-Saharan Africa.^1^ Mother-to-child transmission (MTCT) of HIV is the main source (90%) of HIV acquisition among children; without any intervention, up to 45% of pregnant or breastfeeding HIV-infected women transmit HIV to their children.^2,3^

With political commitment in many countries, integration of HIV-related programmes into routine healthcare, and availability and use of potent and combined antiretroviral drugs, prevention of MTCT of HIV (PMTCT) services have remarkably reduced the number of new paediatric HIV infections.^4^ However, the global goal of reducing MTCT to 2% or less has been reached in only a few lower- and middle-income countries.^5^ It is plausible that the slow progress towards elimination of MTCT (eMTCT) is because of the low capacity of health systems to (1) translate the political will into real life action, (2) and implement scientific advancements – early identification of HIV-positive pregnant women and initiation of potent antiretroviral treatment – with fidelity.^6,7,8^

Global health institutions (World Health Organization [WHO], Joint United Nations Programme on HIV/AIDS [UNAIDS], United Nations Children’s Fund [UNICEF]) and many national programmes have called for eMTCT at the population level. There has been a focus on 21 countries that account for 90% of the burden of new HIV infections^6,7^; Kenya is one of the 21 priority countries. By 2021, 14 countries were certified as having eliminated MTCT, all in the Caribbean, South America, Eastern Europe, and Asia. Apart from Botswana, which is on the path to eMTCT, no sub-Saharan African country has reached eMTCT.^8^ In 2020, 13 (including Kenya) of the 21 priority countries in Africa had an overall MTCT rate of 10% or higher; about half of this transmission occurred during breastfeeding.^1^ Available data suggest that in Eastern and Southern Africa, the majority of MTCT happened with women with incident HIV infections and those who dropped out of care; in Western and Central Africa, MTCT occurred mainly through women who did not receive antiretroviral therapy (ART).^1,8^

Kenya – one of the 21 priority countries – officially launched its national PMTCT programme in 2002.^9^ Since then, Kenya has been implementing PMTCT programmes with encouraging progress. All pregnant women are offered screening for HIV infection and those with HIV infection are put on ART. After birth, their HIV-exposed children receive prophylactic antiretrovirals (ARVs); the recommended feeding option is exclusive breastmilk for the first 6 months. Early infant diagnosis (EID) is offered as early as 2 months postpartum using HIV deoxyribonucleic acid (DNA) polymerase chain reaction (PCR). The 18-month MTCT rate in Kenya has trended downwards, from 16% in 2012 to 11.4% in 2017.^10,11^ However, these figures are too far above the threshold of 5% to satisfy the eMTCT criteria.^12^

In Kenya, data from 205 health facilities supported by FHI 360, in six rural and peri-urban counties, showed an MTCT rate of 7.4% in 2017 and 2.1% in 2020. Our objective was to analyse factors that potentially correlate to the change in MTCT rates between the two time points. We analysed PMTCT service data to compare mother–baby pairs where MTCT occurred with pairs where MTCT did not occur to identify predictors of MTCT at these two time points.

We believe that the analysis will be useful to inform efforts towards eMTCT.

## Research methods and design

### Setting

In Kenya, PMTCT is a routine intervention that has been integrated into reproductive, maternal, newborn, and child healthcare (RMNCH) since 2014. Since the launch of PMTCT services in 2009, Kenya PMTCT guidelines have been continuously updated in line with WHO recommendations.^13^

Altogether, in six (of Kenya’s 47 counties) counties, we analysed data from 205 health facilities (primary health centres and hospitals): 19 facilities in Baringo County, 29 in Kajiado County, 28 in Laikipia County, 106 in Nakuru County, 14 in Narok County, and 9 in Samburu County.

These health facilities were purposely selected as they received technical, logistic, and financial support from the United States Agency for International Development (USAID) (Award number AID-615-A-16-00011) through FHI 360, an international non-governmental organisation. The uptake of PMTCT interventions had been better in these facilities than in typical health facilities in Kenya; in 2017, more than 95% of identified HIV-positive pregnant women started on or were receiving ART compared to 76% overall in Kenya.^11^ The FHI 360 support to the health system in Kenya focused on geographic expansion of PMTCT and continuous upgrades of interventions.

The technical, logistic, and financial support aimed to remove systems- and service-level obstacles to access. The assistance included training, job aids, supply chain support, health management and information systems strengthening, support to laboratory systems for blood sample referral, and communication of results to service points.

### Design

This was a retrospective, cross-sectional, exploratory analysis of data collected during routine programme implementation at two time points (in 2017 and 2020). The 3-year span was chosen based on the availability of analysable data. Mother-to-child transmission rates were examined at both time points in relation to other programmatic factors.

### Study population

The population for this study consisted of (1) all HIV-positive mothers and their HIV-exposed children that sought PMTCT services from the selected health facilities between October 2016 and September 2017 and (2) all those who sought services at the same facilities between October 2019 and September 2020.

### Data analysis

#### Data extraction procedures

The data for this analysis originated from a centralised database maintained by the Government of Kenya that captures routine service data. To ensure the high quality and accuracy of data collected, all FHI 360-supported facilities complete a data verification and improvement (DVI) process every 3 months. During DVI, data accuracy is determined, root causes of inaccurate data are identified, data are corrected, and specific prevention recommendations are formulated. A monitoring and evaluation (M&E) officer supported by FHI 360 extracted data from October 2017 to September 2018 – considered as fiscal year (FY) 2017 – and again from October 2020 to September 2021 – considered as FY 2020 – from the antenatal and postnatal registers data captured in the centralised database. The M&E officer removed patients’ names from the extracts; the only unique identifier was patient identification (ID).

Once the de-identified dataset was prepared, it was sent via secured electronic channel to a data analyst for cleaning and analysis. It is possible, although unlikely, the same HIV-positive woman may be represented in both data extracts. A woman could have given birth in 2017 and in 2020, and we cannot cross-reference her inclusion in both extracts as the structure of patient IDs changed over the 3 years. However, it can be verified that no individual woman was represented twice in the same FY as all duplicate IDs were removed, and none of those removed had birth data on the 2 different years.

#### Variables extracted

The structure of the centralised database and some of the key indicators collected changed over the 3-year period (per national guidance). As such, our analysis was limited to indicators that were common to both time points. The one exception was the type of ART regimen delivered to mothers, which was only captured for FY 2020. These indicators were:

- county
- sub-county
- facility name
- infant’s HIV status from the first test
- mother’s age
- infant age at first PCR test
- whether the mother was on ART at time of delivery. At the second time point (2020), the type of ART is also captured (Note: This was only captured at the second time point as certain regimens, such as tenofovir disoproxil fumarate [TDF]-based regimens, were not available in 2017)
- whether the mother was enrolled in HIV comprehensive care and counselling (CCC)
- whether the mother had a recent viral load test recorded
- if viral load test, result
- receipt of infant ARV prophylaxis or not
- exclusively breastfed.

#### Data analysis methods

Analysis of the data was completed in Stata 16.0. Infant HIV status was examined at each time point and by county. Differences in the factors described above by infant HIV status are presented as means for continuous variables and percentages for proportions. Bivariate associations between infant HIV status and each factor were assessed through an unweighted logistic regression analysis. Those associated statistically (defined as *p* < 0.05) are reported. The interaction terms between maternal receipt of ART and infant receipt of prophylaxis, maternal receipt of ART and exclusive breastfeeding, as well as infant receipt of prophylaxis and exclusive breastfeeding were also considered. Possible variations in service delivery were accounted for through dummy variables for each of the counties. A variable for time was also created to indicate whether a case in the dataset came from 2017 or 2020.

An unweighted, multivariable, logistic regression equation was used to model infant HIV status at the first test. The full model developed is descriptive and does not predict or infer causality. The factors that are significant at the *p* < 0.05 level were interpreted as being associated with the outcome of interest. The entire analysis was repeated using data only from sub-counties that contributed data at both time points. This eliminated data from Samburu and Narok.

### Ethical considerations

Ethical approval was granted by the Scientific and Ethical Review Unit (SERU) of the Kenya Medical Research Institute (KEMRI) (KEMRI/RES/7/3/1) as well as by the Protection of Human Subjects Committee of FHI 360, North Carolina, United States. The committees determined the study was non-human subject research.

## Results

### Overall findings

The proportion of children testing positive for HIV at their first test was 7.3% in 2017 compared to 2.1% in 2020 (Table 1). There was great variation across counties in the proportion of children testing positive in 2017, from 3.1% (*n* = 32) in Baringo to 100% in Kajiado (*n* = 13). By 2020, FHI 360-supported facilities were close to reaching the goal of less than 2% MTCT, although only one county, Laikipia, met that goal.

**TABLE 1:** Percentage of human immunodeficiency virus-positive infants from all counties contributing data at any time point (2017 or 2020).

Counties	Percentage of HIV-positive infants at first test	
	FY 2017	FY 2020
Overall	7.3% (out of 593)†	2.1% (out of 6386)
Baringo	3.1% (out of 32)	2.6% (out of 348)
Kajiado	100% (out of 13)	2.2% (out of 1526)
Laikipia	8.9% (out of 56)	1.4% (out of 629)
Nakuru	4.8% (out of 414)	2.1% (out of 3772)
Narok	5.1% (out of 78)	NA
Samburu	NA	5.4% (out of 111)

HIV, human immunodeficiency virus; FY, fiscal year; NA, not available.

† , These are ‘out of’ infant born to HIV-positive mothers.

When the analysis was restricted to those sub-counties that contributed data at both time points, the overall reduction from FY 2017 to FY 2020 was similar. There was similar variation among the counties in 2017, but less in 2020, with MTCT ranging from 1.4% to 2.6% (Table 2).

**TABLE 2:** Percentage of human immunodeficiency virus-positive infants among the sub-counties contributing data at both time points (2017 and 2020).

Counties	Percentage of HIV-positive infants at first test	
	FY 2017	FY 2021
Overall	7.7% (out of 507)	2.0% (out of 5300)
Baringo	3.1% (out of 32)	2.6% (out of 313)
Kajiado	100% (out of 13)	2.0% (out of 1144)
Laikipia	10.4% (out of 48)	1.4% (out of 629)
Nakuru	4.8% (out of 414)	2.1% (out of 3214)
Narok	NA	NA
Samburu	NA	NA

HIV, human immunodeficiency virus; FY, fiscal year; NA, not available.

### General factors associated with mother-to-child transmission

Some of the factors affecting MTCT are shown in Table 3. From 2017 to 2020, the average age of mothers increased from 28.4 to 30.1 years. At each time point, HIV-positive infants were older than HIV-negative infants (FY 2017: 7.1 months compared to 2.2; FY 2020: 6.9 months compared to 5.9). Furthermore, the average age of the infants at their first test increased from 2017 to 2020, from 2.5 to 5.9 months. Between 2017 and 2020, laboratories capable of performing tests for EID faced more and more logistic issues including frequent stockouts of reagents and episodes of equipment breakdown. The proportion of HIV-positive mothers on ART increased from 92% in 2017 to 98.6% in 2020.

**TABLE 3:** Percentage of human immunodeficiency virus-positive infants by factor from all sub-counties contributing data at any time point (2017 or 2020).

Characteristics of mothers and infants	FY 2017			FY 2020		
	HIV −	HIV +	Total	HIV −	HIV +	Total
Mother’s mean age (in years)	28.3	28.8	28.4	30.2	28.3	30.1
Infant’s mean age at test (in months)	2.2	7.1	2.5	5.9	6.9	5.9
Mother enrolled in CCC (%)	96.0	81.4	94.9	21.1	14.1	21.0
Mother on ART (%)	96.5	33.3	92.0	98.9	85.6	98.6
Mother on TDF-based ART (%)	NA†	NA	NA	94.6	80.8	94.3
**Mother viral load (during pregnancy)**						
Percent recorded and suppressed‡	58.0	9.3	54.5	38.9	11.1	38.3
Percent not recorded	40.6	88.4	44.0	58.2	84.4	58.8
Percent recorded and not suppressed	1.6	2.3	1.7	2.9	4.4	3.0
Infant given prophylaxis (%)	96.0	37.2	91.7	95.6	66.7	95.0
Infant exclusively breastfed (%)	88.7	41.9	85.3	61.2	51.9	61.0

HIV −, HIV-negative; HIV +, HIV-positive; FY, fiscal year; NA, not available; CCC, comprehensive care and counselling; TDF, tenofovir disoproxil fumarate; ART, antiretroviral therapy.

† , TDF-based ART regimen was not available (NA);

‡ , Suppression was defined as < 1000 copies/mL.

### Antiretroviral therapy-related factors associated with mother-to-child transmission

The TDF-based ART regimen did not become available until 2020. In 2020, 94.6% of mothers of HIV-negative infants were taking a TDF-based regimen, compared to 80.8% of mothers of positive infants. Maternal viral load was not consistently recorded. The proportion not recorded increased from 44.0% in 2017 to 58.8% in 2020. The proportion of infants receiving prophylaxis increased from 91.7% in 2017 to 95.0% in 2020. The proportion of infants who were reported to be exclusively breastfed declined from 85.3% in 2017 to 61.0% in 2020.

When the analysis was restricted to just those sub-counties that were part of the facilities contributing data at both time points, very similar patterns emerged (Table 4).

**TABLE 4:** Percentage of human immunodeficiency virus-positive infants by factor from all sub-counties contributing data at both time points (2017 and 2020).

Characteristics of mothers and infants	FY 2017			FY 2020		
	HIV –	HIV +	Total	HIV –	HIV +	Total
Mother’s mean age (in years)	28.4	28.9	28.4	30.2	28.4	30.1
Infant’s mean age at test (in months)	2.2	7.1	2.5	5.8	6.9	5.8
Mother enrolled in CCC (%)	97.0	87.2	96.3	21.7	14.8	21.5
Mother on ART (%)	96.8	34.2	92.1	98.9	82.4	98.5
Mother on TDF-based ART (%)	0.0	0.0	0.0	94.3	77.5	94.0
**Mother viral load (during pregnancy or lactating period)**						
Percent recorded and suppressed†	59.4	10.3	55.6	40.8	12.0	40.2
Percent not recorded	39.7	87.2	43.4	56.3	83.3	56.9
Percent recorded and not suppressed	0.9	2.6	1.0	2.9	4.6	2.9
Infant given prophylaxis (%)	96.8	38.5	92.3	95.7	65.7	95.1
Infant exclusively breastfed (%)	89.1	38.5	85.2	61.6	53.7	61.5

HIV –, HIV-negative; HIV +, HIV-positive; FY, fiscal year; CCC, comprehensive care and counselling; TDF, tenofovir disoproxil fumarate; ART, antiretroviral therapy.

† , Suppression was defined as < 1000 copies/mL.

In the univariate regression analysis, time point (2017 or 2020), mother’s age, infant age at test, maternal receipt of ART, maternal receipt of TDF ART, infant receipt of ARVs, infant exclusively breastfed, and all the interaction terms were associated with the outcome (data not shown). In the univariate regression analysis, which was restricted to sub-counties contributing data at both time points, time, maternal age, age of infant at first test, maternal receipt of ART, maternal receipt of TDF-based ART, maternal viral load, child receipt of prophylaxis, exclusive breastfeeding, and all the interaction terms were independently associated with the outcome.

### Findings from the full model analysis

In the full model, the factors significantly associated with the outcome were time points (odds ratio [OR]: 0.148; *p* = 0.000), mother’s age in years (OR: 0.965, *p* = 0.036), infant age at first test (OR: 1.067; *p* = 0.011), mother receipt of ART (OR: 0.137; *p* < 0.000), and maternal viral load (OR: 2.982; *p* < 0.000) (Table 5). The analysis was repeated and restricted to only the sub-counties contributing data at both time points; overall, the results were similar; however, the county of Kajiado became significant in the updated model, as did the interaction term for mother and infant receipt of ARVs (OR: 0.228; *p* = 0.04). Mother’s age was no longer a significant factor.

**TABLE 5:** Multivariate analysis of factors predicting human immunodeficiency virus-positive results in human immunodeficiency virus-exposed infants.

Factor (*reference*)	Infant HIV-positive – Full model		Infant HIV-positive – Full model restricted†	
	AOR	*p*	AOR	*p*
Time (0 = 2017; 1 = 2020)	**0.148**	< 0.000***	**0.163**	< 0.000***
Samburu (0 = no; 1 = yes)	4.094	0.060*	NA	-
Narok (0 = no; 1 = yes)	0.332	0.209	NA	-
Nakuru (0 = no; 1 = yes)	1.292	0.573	1.398	0.506
Laikipia (0 = no; 1 = yes)	0.814	0.736	0.900	0.870
Kajiado (0 = no; 1 = yes)	2.208	0.105	**2.900**	0.050**
Mother’s age in years (continuous)	**0.965**	0.036**	0.970	0.110
Infant age at test in months (continuous)	**1.067**	0.011**	**1.097**	0.001***
Mother in CCC (0 = no; 1 = yes)	0.599	0.067*	0.629	0.134
Mother received ART (0 = no; 1 = yes)	**0.137**	< 0.000***	**0.178**	0.004**
Mother received TDF ART (0 = no/NA; 1 = yes)	1.631	0.297	1.312	0.570
Maternal viral load (0 = suppressed; 1 = not taken; 2 = not suppressed)	**2.982**	< 0.000***	**3.064**	< 0.000***
Infant receipt of ARV (0 = no; 1 = yes)	0.393	0.161	0.513	0.322
Infant exclusively breastfed (0 = no; 1 = yes)	0.397	0.085*	0.404	0.112
Interaction mother and/or child receipt of ARV	0.362	0.144	**0.228**	0.040**
Interaction mother receipt of ART and exclusive breastfeeding	1.101	0.890	1.351	0.685
Interaction infant receipt of ART and exclusive breastfeeding	2.223	0.237	2.393	0.227

HIV, human immunodeficiency virus; AOR, adjusted odds ratio; CCC, comprehensive care and counselling; TDF, tenofovir disoproxil fumarate; ART, antiretroviral therapy; ARV, antiretroviral; NA, not available.

† , Restricted to the counties that contributed data at both time points.

* , *p* < 0.05;

** , *p* < 0.01;

*** , *p* < 0.001.

## Discussion

Our analysis and findings in selected health facilities in Kenya mirror the estimates showing the downward trend of MTCT rates observed worldwide and in Kenya in particular. As per global estimates, the steady reduction of MTCT rates parallels the increase in percentage of pregnant women living with HIV being identified and initiated on ART.^5^ Our analysis also showed that between the two time points (2017 and 2020), an increased number of women living with HIV had been covered by the PMTCT programmes; this mirrors the upward trend of ART uptake among pregnant women living with HIV reported by the Kenya Ministry of Health.^11^

The merit of this study is that it presents predictors that could help explain the drastic reduction of MTCT in a 3-year span. By doing so, the findings open some windows on what HIV programmes can do to accelerate the pace towards elimination of MTCT in similar contexts in Kenya and other countries. Many other manuscripts have explored the question, and overall, have found similar factors associated with MTCT.^14,15^ It has been established that the mother’s viral load is the most critical factor in predicting occurrence of MTCT.^16,17,18^ In line with this, our multivariate analysis also showed that the mother’s viral load was the strongest predictive factor associated with occurrence of MTCT.

The second-strongest correlate to the MTCT rate is timing; our multivariate analysis found that mothers with HIV had lower odds of transmitting HIV to their children at time two (2020) than at time one (2017). The timing should be interpreted as a change in known factors associated with MTCT. Between 2017 and 2020, a higher proportion of pregnant women living with HIV had been initiated on ART and a TDF-based ART had been introduced. Another potential predictor explained by the timing is the increasing proportion of pregnant women living with HIV already identified as living with HIV and likely on ART at their first antenatal visit. As per the existing literature, HIV-positive women who become pregnant on ART have significantly lower MTCT rates than women who initiated highly active antiretroviral therapy (HAART) during pregnancy.^18,19,20^ In FHI 360-supported antenatal clinics, service data (not presented here) show a growing proportion of pregnant women living with HIV already on ART at their first visit. This observation mirrors the global trend.^8^

As described earlier, the mean age of children at the time of first HIV EID was 2.5 months at time one (2017) and 5.9 months at time two (2020). Many programmes found that testing older HIV-exposed infants is associated with higher MTCT rates.^21^ From our analysis, finding a lower MTCT rate at time two, despite the older age at early infant diagnostic, further confirms the observed improvement of PMTCT services.

### Limitations

Our analysis did not consider some qualitative aspects that can affect uptake of PMTCT interventions such as stigma and discrimination, male partner involvement, and understanding of PMTCT interventions.^22,23,24,25,26^ Maternal viral load was not consistently recorded at time two (2020), thus we could not demonstrate that mothers were virally suppressed at a higher proportion when compared to time one (2017). From our data and analysis, association between maternal viral load and MTCT rate should be interpreted with caution.

Another limitation is the shift in the approach in offering of HIV-related services; in 2017, HIV-related services were more centralised and less integrated than in 2020. It is possible that in 2017 there was a bias in selection of infants for early HIV diagnostic. In Narok county in 2017, all 13 HIV-exposed children tested positive (Table 1); this yield of HIV-positive results should be considered with caution.

Many other factors may be correlated to the change in the rate of MTCT between these two time points. These other factors could be related to the health system or proportion of HIV-positive pregnant women already on ART at their first visit. Unfortunately, we could not examine those potential factors using available retrospective routine service data as they are limited. Further research should examine health system-related factors more thoroughly. Our findings might be better interpreted if compared to similar studies that consider these other potential factors.

Our analysis did not take into account the final outcome of HIV-exposed infants – their final HIV status and/or their survival. The data needed to perform this analysis were not available because the health management information system for routine service did not record them. We would also like to caution generalising our findings to the entire Kenya given that the studied facilities were purposely selected.

## Conclusion

Encouragingly, between 2017 and 2020, in 77 health facilities in semi-urban and rural Kenya, the rate of MTCT of HIV was reduced from 7.3% to 2.1%. Between the two time points, our analysis found that a higher proportion of pregnant women living with HIV received ART. In addition, unlike in 2017, in 2020, TDF was the backbone of the ART regimen of the PMTCT intervention. Another plausible explanation was length of time in ART programmes as a growing proportion of pregnant women living with HIV were registered in maternal health and PMTCT services and already on ART. This second explanation would require a more rigorous study. We suggest that this type of analysis should be part of PMTCT programme implementation to ensure that progress (or lack of it) is continuously measured, lessons are learned, and improvements are made. From the study limitations, this analysis is a call for good quality data collection, and possibly addition of custom indicators to make future analysis robust.

## References

[1] UNAIDS. AidsInfo global data on HIV epidemiology and response. Geneva: UNAIDS; 2021.

[2] World Health Organization. Global HIV program, mother-to-child transmission of HIV, triple elimination initiative [homepage on the Internet]. WHO; 2020 [cited 2022 Jan 10]. Available from: https://www.who.int/teams/global-hiv-hepatitis-and-stis-programmes/hiv/prevention/mother-to-child-transmission-of-hiv

[3] De Cock KM, Fowler MG, Mercier E, et al. Prevention of mother-to-child HIV transmission in resource-poor countries: Translating research into policy and practice. JAMA. 2000;283(9):1175–1182. 10.1001/jama.283.9.1175 10703780

[4] UNICEF. Elimination of mother-to-child transmission, current status and progress [homepage on the Internet]. UNICEF; 2021 [cited 2022 Jan 10]. Available from: https://data.unicef.org/topic/hivaids/emtct/#

[5] HIV estimates for children dashboard [homepage on the Internet]. UNICEF; 2021 [cited 2022 Jan 10]. Available from: https://data.unicef.org/resources/hiv-estimates-for-children-dashboard/

[6] UNAIDS. 2016 United Nations political declaration on ending AIDS sets world on the fast-track to end the epidemic by 2030 [homepage on the Internet]. UNAIDS; 2016 [cited 2022 Jan 11]. Available from: https://www.unaids.org/en/resources/presscentre/pressreleaseandstatementarchive/2016/june/20160608_PS_HLM_PoliticalDeclaration

[7] UNAIDS. Start free stay free AIDS free – 2017 progress report. Geneva: UNAIDS; 2017.

[8] Luo C. State of the art eMTCT. Proceedings of the INTEREST Conference; 2022 May 10–13; Kampala.

[9] Kenya Ministry of Health. Guidelines for prevention of mother-to-child transmission (PMTCT) of HIV/AIDS in Kenya. Nairobi: Kenya Ministry of Health; 2012.

[10] Kenya Ministry of Health and National AIDS Control Council. Kenya AIDS response: Progress report 2016. Nairobi: Kenya Ministry of Health and National AIDS Control Council; 2016.

[11] Kenya Ministry of Health and National AIDS Control Council. Kenya AIDS response: Progress report 2018. Nairobi: Kenya Ministry of Health and National AIDS Control Council; 2018.

[12] World Heath Organization. Global guidance on criteria and process for validation: Elimination of mother-to-child transmission of HIV and syphilis [homepage on the Internet]. 2nd ed. WHO; 2017 [cited 2022 Jan 11]. Available from: https://www.who.int/publications/i/item/9789240039360

[13] Kenya Ministry of Health. Towards the elimination of mother-to-child transmission of HIV and keeping mothers alive [homepage on the Internet]. Kenya Ministry of Health; 2009 [cited 2022 Jan 17]. Available from: http://guidelines.health.go.ke:8000/media/Strategic_Framework_for_EMTCT_in_Kenya-21.pdf

[14] Nduati EW, Hassan AS, Knight MG, et al. Outcomes of prevention of mother to child transmission of the human immunodeficiency virus-1 in rural Kenya – A cohort study. BMC Public Health. 2015;15:1008. 10.1186/s12889-015-2355-4 26433396 PMC4592570

[15] Okoko NA, Owuor KO, Kulzer JL, et al. Factors associated with mother to child transmission of HIV despite overall low transmission rates in HIV-exposed infants in rural Kenya. Int J STD AIDS. 2017;28(12):1215–1223. 10.1177/0956462417693735 28181860

[16] Landes M, Van Lettow M, Nkhoma E, et al. Low detectable postpartum viral load is associated with HIV transmission in Malawi’s prevention of mother-to-child transmission programme. J Int AIDS Soc. 2019;22(6):e25290. 10.1002/jia2.25290 31180186 PMC6556977

[17] Mock PA, Shaffer N, Bhadrakom C, et al. Maternal viral load and timing of mother-to-child HIV transmission, Bangkok, Thailand. Bangkok Collaborative Perinatal HIV Transmission Study Group. AIDS. 1999;13(3):407–414. 10.1097/00002030-199902250-00014 10199232

[18] Myer L, Phillips TK, McIntyre JA, et al. HIV viraemia and mother-to-child transmission risk after antiretroviral therapy initiation in pregnancy in Cape Town, South Africa. HIV Med. 2017;18(2):80–88. 10.1111/hiv.12397 27353189

[19] Hoffman RM, Black V, Technau K, et al. Effects of highly active antiretroviral therapy duration and regimen on risk for mother-to-child transmission of HIV in Johannesburg, South Africa. J Acquir Immune Defic Syndr. 2010;54(1):35–41. 10.1097/QAI.0b013e3181cf9979 20216425 PMC2880466

[20] Chibwesha CJ, Giganti MJ, Putta N, et al. Optimal time on HAART for prevention of mother-to-child transmission of HIV. J Acquir Immune Defic Syndr. 2011;58(2):224–228. 10.1097/QAI.0b013e318229147e 21709566 PMC3605973

[21] Dakum P, Tola M, Iboro N, et al. Correlates and determinants of early infant diagnosis outcomes in North-Central Nigeria. AIDS Res Ther. 2019;16(1):27. 10.1186/s12981-019-0245-z 31521170 PMC6744629

[22] Mutabazi JC, Gray C, Muhwava L, et al. Integrating the prevention of mother-to-child transmission of HIV into primary healthcare services after AIDS denialism in South Africa: Perspectives of experts and health care workers – A qualitative study. BMC Health Serv Res. 2020;20(1):582. 10.1186/s12913-020-05381-5 32586318 PMC7318762

[23] Berlacher M, Mercer T, Apondi EO, Mwangi W, Were E, McHenry M. Integrating prevention of mother-to-child transmission of HIV care into general maternal child health care in Western Kenya. Int J MCH AIDS. 2021;10(1):19–28. 10.21106/ijma.429 33442489 PMC7792744

[24] Hampanda K, Helova A, Odwar T, et al. Male partner involvement and successful completion of the prevention of mother-to-child transmission continuum of care in Kenya. Int J Gynaecol Obstet. 2021;152(3):409–415. 10.1002/ijgo.13442 33108671 PMC7902296

[25] Philemon RN, Mmbaga BT, Bartlett J, et al. Do women enrolled in PMTCT understand the recommendations: A case study from Kilimanjaro. Patient Prefer Adherence. 2021;15:1301–1309. 10.2147/PPA.S307847 34163147 PMC8216065

[26] Prudden HJ, Hamilton M, Foss AM, et al. Can mother-to-child transmission of HIV be eliminated without addressing the issue of stigma? Modeling the case for a setting in South Africa. PLoS One. 2017;12(12):e0189079. 10.1371/journal.pone.0189079 29220369 PMC5722282

